# Identifying Visceral Kaposi Sarcoma (KS): A Responsibility to Avoid Anchoring on the Diagnosis of a Crohn’s Flare

**DOI:** 10.7759/cureus.34986

**Published:** 2023-02-14

**Authors:** Emily S Seltzer, Shabari M Shenoy, Bo Hyung Yoon, Frederick Rozenshteyn, Kimberly Cavaliere

**Affiliations:** 1 Internal Medicine, Mount Sinai West and Morningside, New York, USA; 2 Gastroenterology, Mount Sinai Beth Israel, West and Morningside, New York, USA

**Keywords:** colonoscopy, crohn’s disease (cd), disseminated kaposi sarcoma, hiv associated malignancies, inflammatory bowel disease

## Abstract

The initial evaluation of reported inflammatory bowel disease (IBD) should include an assessment for immunosuppression which can broaden the differential diagnosis to include opportunistic infection as well as other processes. Here we present an exceedingly rare case of a patient with a self-reported history of Crohn’s disease presenting with frequent diarrhea presumed to be a Crohn’s flare, however, after further workup was found to have extensive visceral Kaposi sarcoma (KS).

## Introduction

Crohn’s disease (CD) is a T-cell-mediated immune response to an unclear environmental trigger in those genetically predisposed. CD4+ cells are involved in initiating and propagating the disease process of inflammatory bowel disease (IBD) [1-2]. While IBD is an overdrive of the immune system, human immunodeficiency virus (HIV) diminishes the immune system by targeting these same CD4+ lymphocytes [3]. Having concomitant IBD and HIV is rare with a prevalence of about 0.4% [4]. The reason for this is unknown, however, it has been hypothesized that the decline in CD4+ count in HIV promotes remission of IBD by decreasing the inflammatory response [5]. In this article, we describe a patient with a reported diagnosis of CD presenting with symptoms consistent with previous IBD flares. Upon further evaluation, our patient was discovered to be HIV positive with multiple opportunistic infections (OI) including visceral Kaposi sarcoma (KS) of the gastrointestinal tract.

## Case presentation

**Figure 1:**
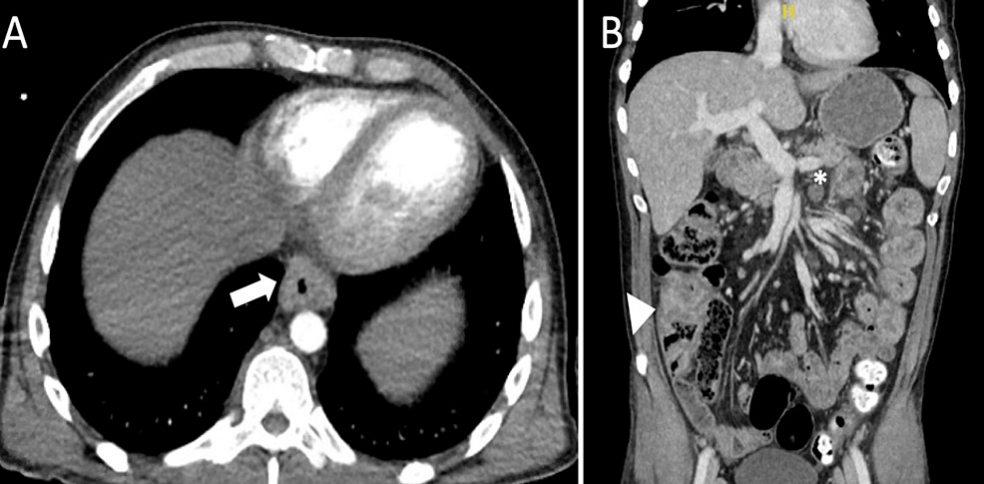
Computed aomography angiography abdomen and pelvis on initial presentation. Computed tomography angiography with (A) mural thickening in the mid and distal esophagus (arrow) and (B) new mesenteric adenopathy (asterisk) and localized wall thickening of ascending colon (arrowhead).

A 45-year-old male with a reported history of CD off therapy, presented with six months of worsening weakness, abdominal pain, bloody diarrhea (>10 episodes most days), dysphagia to solids, and a 27 lbs weight loss. On initial presentation, he was afebrile, tachycardic with exam significant for multiple violaceous plaques on his left leg and left lower quadrant abdominal tenderness. Noteworthy labs included hemoglobin 9 g/dL (baseline 12 g/dL), normal leukocyte count, C-reactive protein 22 mg/L, and erythrocyte sedimentation rate 100 mm/h. Computed tomography (CT) angiography revealed mild mural thickening in the mid and distal esophagus, single enlarged hilar lymph node, mesenteric adenopathy, splenomegaly, and localized wall thickening of the ascending colon (Figure 1A,B).

**Figure 2:**
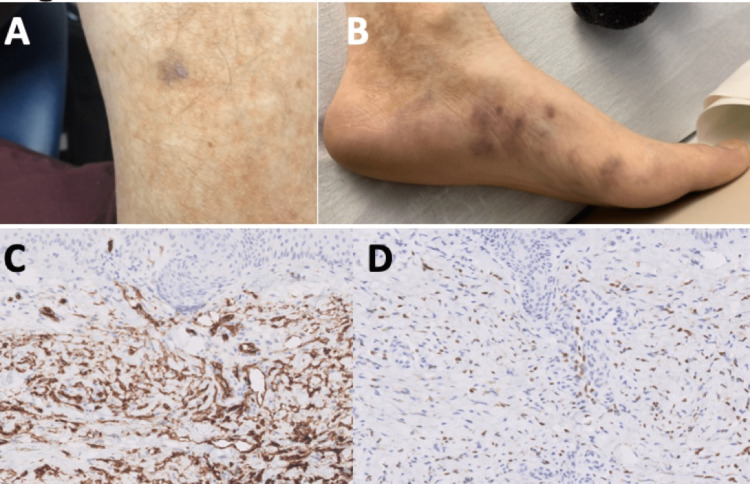
Lower extremity violaceous lesions and immunohistochemistry of biopsy. KS skin lesions on the left (A) leg and (B) foot, which were biopsied displaying positive (C) CD34, highlighting the vascular spaces, and (D) human herpes virus 8, highlighting endothelial cell nuclei

On initial consultation, review of previous colonoscopies and pathology results demonstrated chronic active colitis in the left and right colon, including cryptitis and neutrophils loose in the lamina propria without glandular architectural disorganization, consistent with IBD but not definitive for Crohn’s. We requested stool studies to rule out infectious etiology. Upon questioning, we discovered his lower extremity lesions were recently diagnosed as KS, confirmed with CD34 and human herpesvirus type 8 (HHV8) (Figure 2A-D), so we suggested HIV testing. Prior to the recommended labs resulting, the patient developed a fever of 101.3°F, heart rate of 115, platelets 60 x 103/mm3, leukocytes 3.3 K/μL, and ceftriaxone and metronidazole were empirically started. A second CT scan to assess interval change with possible abscess or perforation, given the new developments and persistent symptoms, remained unchanged. Eventually, blood cultures, Clostridium difficile, and gastrointestinal pathogens panel resulted as negative and fecal calprotectin was 761 μg/g. Initiation of high-dose glucocorticoid therapy was deferred given the unclear infectious etiology and suspicion for new-onset HIV despite the fact the patient had been screened yearly using the HIV-1/2 antigen/antibody combination immunoassay test, which had been non-reactive. 

The HIV results were positive with a CD4+ count of 58 cells/mm3 and viral load >1,000,000 copies/mL. Given his clinical course, more objective information was required to decide the appropriate treatment course, thus an upper endoscopy and colonoscopy were performed.

**Figure 3:**
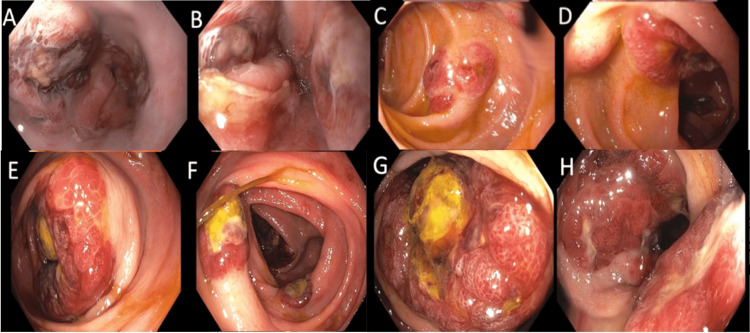
Upper endoscopy and colonoscopy. Endoscopy/colonoscopy of multiple 10-50 mm violaceous nodules throughout the (A) distal esophagus, (B) gastroesophageal junction, (C, D) second portion of the duodenum (E) ileocecal valve, (F) ascending colon, (G) hepatic flexure, and (H) rectum concerning for KS.

**Figure 4:**
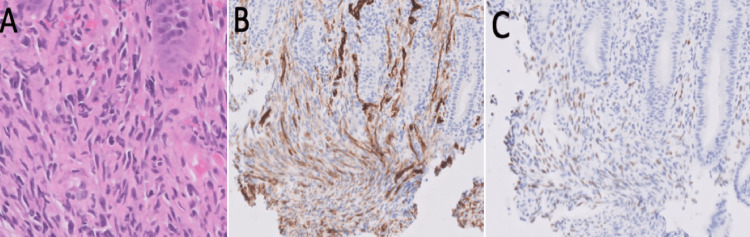
Ileocecal valve lesion biopsy. Ileocecal valve biopsy of one visceral Kaposi sarcoma (KS) lesion displaying (A) colonic mucosa showing lamina propria with atypical spindle cell proliferation with minimal pleomorphism and few extravasated red blood cells, with the atypical spindle cells being positive for (B) CD34 and (c) HHV8 supporting the diagnosis of KS.

Endoscopy demonstrated many 10-50 mm violaceous nodules in the esophagus, second portion of the duodenum (Figure 3A-D), and throughout the colon consistent with KS (Figure 3 E-H). Given the classic appearance and high risk of significant bleeding, only one biopsy of the lesions was taken. Histology confirmed colonic involvement by KS, positive for CD34 and HHV8 on immunohistochemistry (Figure 4A-C). Random samples from the left colon revealed lymphoplasmacytic inflammatory infiltrates in the lamina propria extending to the muscularis mucosa, focal hyalinization, and drop out crypts correlating with mixed infectious and IBD.

After endoscopic evaluation, the patient’s KS was staged as tumor 1 (visceral disease), immune system 1 (CD4 <200), and systemic illness 1 (B symptoms and OI). He was started on paclitaxel chemotherapy and completed seven cycles outpatient. HAART was initially held due to risk of immune reconstitution inflammatory syndrome then started a few weeks later through a joint decision-making process. About seven months after initial diagnosis, the patient underwent repeat endoscopy and colonoscopy proving complete eradication of KS from the esophagus and colon.

## Discussion

The KS in patients with HIV and IBD is extremely rare with few cases ever reported [6-8]. Similarly, visceral KS is rarely present at the initial diagnosis of HIV, with an incidence of about 15% [8-11]. Our patient’s presentation demonstrates the risks of anchoring on a previous, likely misdiagnosis of CD, as starting treatment could have been very harmful. After careful review, we found that three years prior to this presentation, the patient had undergone a series of CTs showing proctitis, colonoscopies with non-specific left-sided colitis, and stool studies with fecal calprotectin in the thousands. Having reviewed the previous pathology reports, we were sceptical that he truly had CD. If it was not for this critique of the patient's past diagnosis and our high index of suspicion given his recent diagnosis of cutaneous KS, it is possible that the diagnosis of HIV would have been missed. Missing this diagnosis could have been extremely detrimental to the patient as he was subsequently found to have multiple OIs for which the administration of steroids would have been harmful.

Typically, the diagnosis of an IBD relapse focuses on a patient’s clinical presentation along with inflammatory markers while ruling out stool pathogens, but does not ubiquitously require a colonoscopy to be done before treatment is started [12]. Following that algorithm could have inadvertently led to us starting the patient on high-dose steroids, which has been shown to further progress KS along with other OIs such as candida, cytomegalovirus, hepatitis B and C, and tuberculosis [12-13]. Though, our patient required an endoscopy and colonoscopy given new dysphagia and weight loss, as well as an unclear prior diagnosis of IBD. While it may be reasonable to start corticosteroids, or anti-tumor necrosis factor-alpha for IBD flares in patients receiving HAART or with CD4+ >500, the safety of starting such treatments have not been studied in those with uncontrolled HIV as in our case [6, 14-16].

It is unclear whether the theory regarding low CD4+ counts being protective against IBD remission holds in this instance. Cases supporting this hypothesis exhibited fewer IBD exacerbations, increased time to first relapse, and less use of immunosuppressants [4-5, 8, 17]. One study anticipated that decreased use of immunosuppressives may be due to physicians’ fear of serious infection, which was our reasoning for avoiding steroids early on [8]. Based on the pathology, it is uncertain if our patient was simultaneously suffering from an IBD flare and visceral KS, or if this all could be attributed to his KS alone.

## Conclusions

We share this extremely rare case of visceral KS in a patient with newly diagnosed HIV masquerading as a CD flare to promote broadening physicians’ diagnostic arsenal where the picture of IBD is not clear cut and likely previously misdiagnosed. Specifically, we demonstrate that in such cases, other etiologies should be considered prior to steroid administration. Further studies are required to understand the safety profile in treating IBD relapses in patients with uncontrolled HIV.
